# Phosphoproteomics reveals ALK promote cell progress via RAS/JNK pathway in neuroblastoma

**DOI:** 10.18632/oncotarget.12513

**Published:** 2016-10-07

**Authors:** Kai Chen, Fan Lv, Guofeng Xu, Min Zhang, Yeming Wu, Zhixiang Wu

**Affiliations:** ^1^ Department of Pediatric Surgery, Xinhua Hospital, School of Medicine, Shanghai Jiaotong University, Shanghai, China; ^2^ Division of Pediatric Oncology, Shanghai Institute of Pediatric Research, Shanghai, China

**Keywords:** neuroblastoma, ALK, phosphoproteomics, target therapy, pediatric oncology

## Abstract

Emerging evidence suggests receptor tyrosine kinase ALK as a promising therapeutic target in neuroblastoma. However, clinical trials reveal that a limited proportion of ALK-positive neuroblastoma patients experience clinical benefits from Crizotinib, a clinically approved specific inhibitor of ALK. The precise molecular mechanisms of aberrant ALK activity in neuroblastoma remain elusive, limiting the clinical application of ALK as a therapeutic target in neuroblastoma. Here, we describe a deep quantitative phosphoproteomic approach in which Crizotinib-treated neuroblastoma cell lines bearing aberrant ALK are used to investigate downstream regulated phosphoproteins. We identified more than 19,500—and quantitatively analyzed approximately 10,000—phosphorylation sites from each cell line, ultimately detecting 450–790 significantly-regulated phosphorylation sites. Multiple layers of bioinformatic analysis of the significantly-regulated phosphoproteins identified RAS/JNK as a downstream signaling pathway of ALK, independent of the ALK variant present. Further experiments demonstrated that ALK/JNK signaling could be inactivated by either ALK- or JNK-specific inhibitors, resulting in cell growth inhibition by induction of cell cycle arrest and cell apoptosis. Our study broadly defines the phosphoproteome in response to ALK inhibition and provides a resource for further clinical investigation of ALK as therapeutic target for the treatment of neuroblastoma.

## INTRODUCTION

Neuroblastoma is the most common extracranial solid malignant tumor and accounts for approximately 10% of pediatric cancer related death [1]. Neuroblastomas are highly heterogeneous, with pathological type, disease stage, and MYCN amplification all influencing prognosis [2]. Despite intensive chemotherapy, children with high-risk neuroblastoma have a 5-year event-free survival of less than 31% [3]. Therefore, there is a great need for novel interventions to improve the long-term prognosis.

Recent studies reveal that approximately 8% of primary neuroblastomas have a mutation in the anaplastic lymphoma kinase (ALK) gene [4–7], which encodes a receptor tyrosine kinase. These single amino acid missense substitutions are located in the ALK tyrosine kinase domain and induce the constitutive activation of ALK by auto-phosphorylation, generating an oncogene that drives cancer progression [8]. ALK gene amplification is also involved in neuroblastoma tumorigenesis [8]. Crizotinib (PF-02341066) is approved for the treatment of non-small cell lung cancer (NSCLC) and anaplastic large cell lymphoma (ALCL), which harbor the fusion oncogenes EML4-ALK and NPM-ALK, respectively [9, 10]. Crizotinib effectively reduces ALK kinase activity by competitively binding to the ATP pocket in the kinase domain [11]. Given that mutated ALK shares the same ATP pocket with fusion ALK proteins, neuroblastoma patients with aberrant ALK fusion proteins could benefit from Crizotinib treatment. However, a recent clinical trial revealed that not all neuroblastoma patients with aberrant ALK activation respond to Crizotinib [12]. One proposed explanation for this difference is that membrane-associated point mutated/amplified ALK is found in neuroblastoma, while cytoplasmic ALK fusion proteins are found in other tumors [13]. However, the downstream signaling pathways affected by aberrant ALK activity in neuroblastoma remain unclear, impairing the clinical application of Crizotinib for neuroblastoma treatment.

Mass spectrometry-based phosphoproteomics provides a powerful approach to gain more complete knowledge of a cell signaling network [14]. In this study, we have globally analyzed the effect of Crizotinib treatment on phosphoproteome changes in three representative neuroblastoma cell lines, NB1643, NB1, and SH-SY5Y, which harbor R1275 mutated, amplified, and F1174 mutated ALK, respectively [15]. Approximately 20,000 phosphorylation sites were evaluated in each cell line and quantitative analysis revealed 790, 438, and 497 phosphorylation sites that were significantly regulated by ALK in NB1643, NB1, and SH-SY5Y cell lines, respectively. Bioinformatic analysis identified RAS/JNK as a downstream signaling pathway influenced by ALK in these cell lines—independent of the ALK variant type present. We further showed that ALK/JNK signaling could be inactivated by a specific inhibitor, resulting in cell growth inhibition through induction of apoptosis and cell cycle arrest. Furthermore, we confirmed that inhibition of ALK/JNK signaling led to decreased activity of AP-1. AP-1 regulates numerous cellular processes including cell death, survival, and proliferation [16]. Collectively, this study provides insight into the downstream signaling pathways activated by membrane-bound full-length ALK in neuroblastoma, and demonstrates theoretical support for promoting the clinical use of ALK-specific inhibitors in neuroblastoma patients with aberrant ALK signaling.

## RESULTS

### Identification of the ALK-dependent phosphoproteome

**Figure 1:**
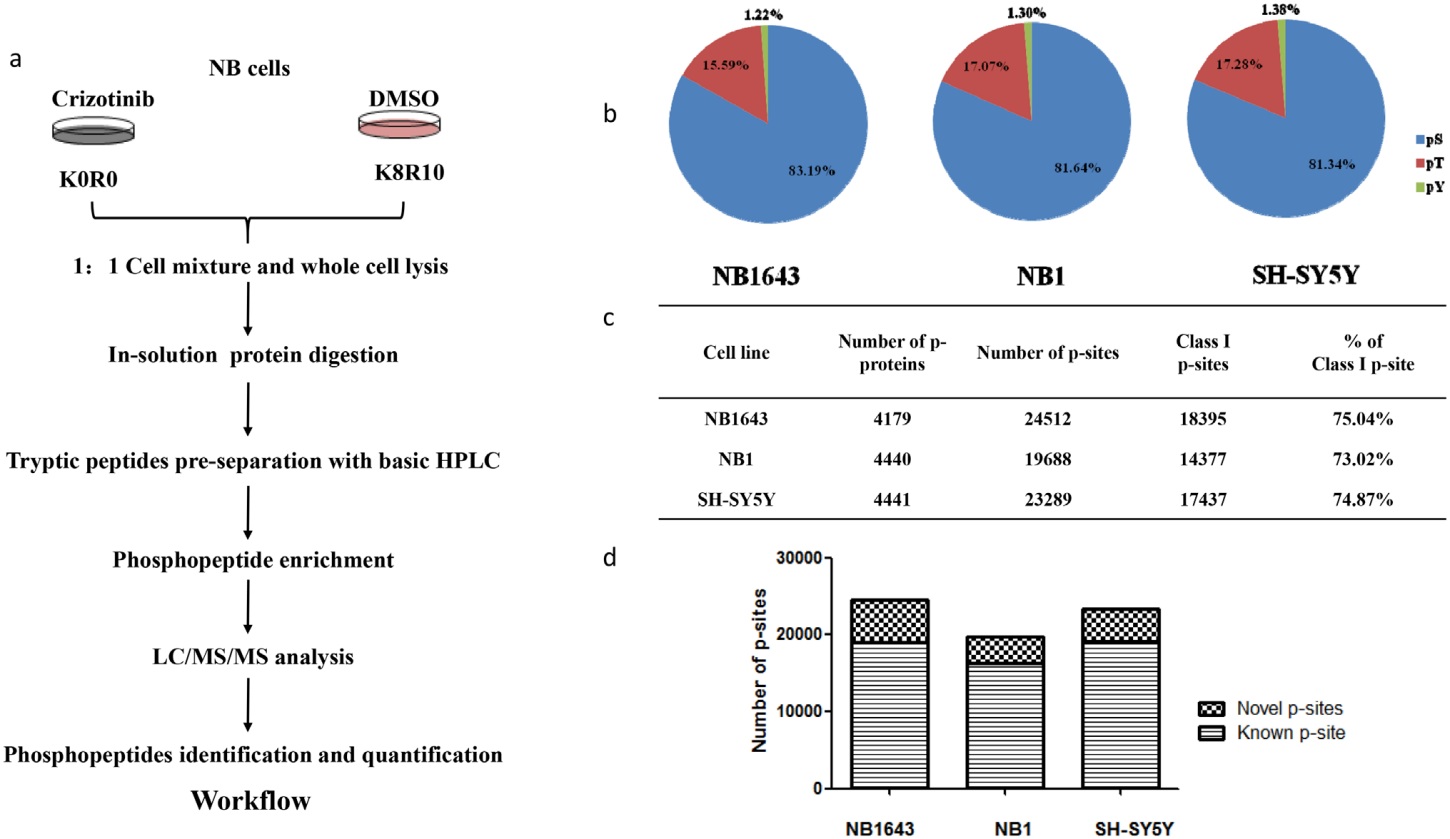
Phosphoproteomics profiling for ALK regulated phosphoproteome in neuroblastoma **a.** Strategy for global analysis ALK regulated phosphoproteome; **b.** Distribution of phosphorylation sites by amino acid; **c.** Number of phosphoprotein and phosphorylation sites identified in three cell lines; **d.** Number of novel phosphorylation sites identified in the indicated cell lines.

We investigated the signaling pathways regulated by ALK in ALK-positive neuroblastoma by comparing the phosphoproteomes of ALK-positive neuroblastoma cell lines following inhibition of ALK by its specific inhibitor Crizotinib (Figure 1a). Dose-response curves were first established for all three cell lines, and consistent with a previous study the IC50 values were 0.5, 0.3, and 1 μM for NB1643, NB1, and SH-SY5Y cells, respectively (Supplemental Figure S1). To enable quantitative comparison of the phosphoproteome, the three cell lines were labeled with stable isotope labeling with amino acids in cell culture (SILAC). Cells labeled “light” were then treated with 10 times the relevant IC50 value for Crizotinib (3–10 μM) for 4 h (Supplemental Figure S1), and the corresponding “heavy” cells were treated with DMSO as control. After drug treatment, cells were harvested, mixed in a 1:1 ratio, and subjected to protein extraction as described previously. After in-solution digestion, tryptic peptides were pre-separated into 20 fractions with high pH RPLC. Phosphopeptides were then enriched with customized IMAC beads [17] and subsequently analyzed using an LTQ-Orbitrap Elite mass spectrometer. Phosphopeptide identification and quantification was achieved using the MaxQuant software package. Each analysis was performed in biological duplicates to enable the confident quantification of phosphorylation sites. Collectively, approximately 20,000 phosphorylation sites from over 4,000 proteins were identified from each cell line, with a false discovery rate of <1% at both the peptide and protein levels. The distribution of pSer, pThr, and pTyr sites was consistent with previous reports (Figure 1b) [18], validating the unbiased enrichment approach we employed. Approximately 75% of phosphopeptides were assigned to a specific residue with high confidence (class I phosphopeptide) (Figure 1c). Comparing our results with those in the PhosphoSitePlus database, we found that approximately 20% of the phosphorylation sites we identified have not been previously reported (Figure 1d and Supplemental Table S1). Specifically, we identified 9,693 phosphorylation sites—79.18% pSer, 21.24% pThr, and 2.58% pTyr—not included in PhosphoSitePlus database. Class I phosphorylation sites account for 54.72% of these novel phosphorylation sites (Supplemental Table S1, Supplemental Figure S3a and Supplemental Figure S3b).

**Figure 2:**
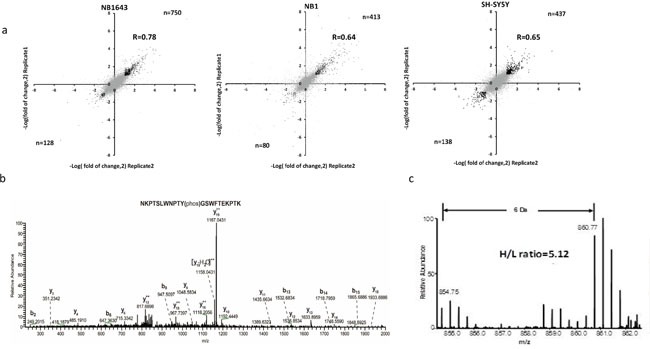
Quantitative analysis of the phosphoproteome upon Crizotinib treatment in ALK positive neuroblastoma **a.** Comparison of pshophorylation sites quantification result between technical replicates, correlation was calculated with Perseus, phosphorylation sites regulated more than 2 times of fold in both replicates were highlighted with black dots; **b.** MS2 spectrum annotation of peptide containing phosphotyrosine (Y1507) on ALK; **c.** MS quantification of Y1507 upon Crizotinib treatment.

Only those phosphopeptides with a greater than two-fold change (either increased or decreased) in both replicates were considered for further analysis (Figure 2a). A complete list of all quantified phosphopeptides is provided in Supplemental Table S1. With this criterion, we found that ALK regulated 790, 438, and 497 phosphorylation sites in NB1643, NB1, and SH-SY5Y, cell lines respectively. Approximately 90% of these regulated phosphorylation sites are Class I phosphorylation sites (Supplemental Figure S3c). Additionally, we observed significantly decreased phosphorylation levels at multiple ALK-regulated sites following Crizotinib treatment of the ALK-amplified neuroblastoma cell line NB1. Furthermore, phosphoryaltion of Y1507 (Y567 in NPM-ALK), a docking site for recruiting SHC1 to activate downstream signaling pathways and promote tumorigenesis [19], was significantly decreased following Crizotinib treatment (Figure 2b).

### Global analysis of ALK-regulated phosphorylation sites

**Figure 3:**
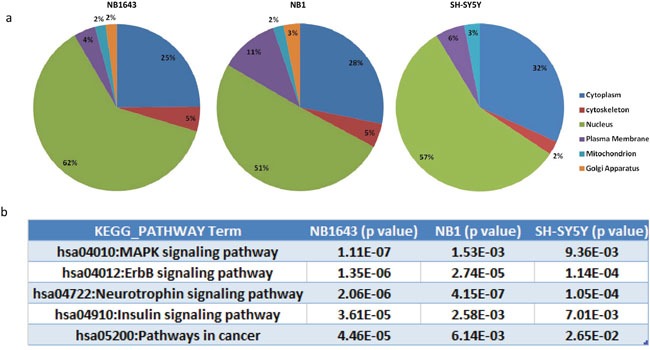
Bioinformatic analysis of the ALK regulated phosphoproteome **a.** Subcellular localization of the phosphorylation proteins regulated by ALK; **b.** Top KEGG pathways enriched by ALK regulated phosphoprotein in three neruoblastoma cell lines.

We next investigated the function of ALK-regulated phosphorylation of proteins in neuroblastoma by applying several layers of bioinformatic analysis. The subcellular localization of signaling molecules plays a vital role in signal transduction, so we first investigated the subcellular localization of ALK-regulated phosphoproteins by querying the LOCATE database [20]. In all three cell lines, over half the interpretable phosphoproteins were present in the nucleus, while approximately 25% were cytoplasmic (Figure 3a). Further query of the DNA binding database revealed that 34, 19, and 16 significantly-regulated phosphoproteins were transcription factors in NB1643, NB1, and SH-SY5Y cell lines, respectively (Supplemental Table S2). This led us to perform UCSC_TFBS (transcription factor binding sites annotation) analysis of the regulated phosphoproteins, revealing a broad range of overrepresented transcription factor complexes. Some of these transcription factor complexes have been implicated in neuroblastoma tumorigenesis, including E2F [21], PAX5 [22], and RFX1 [23]. Our results also identified the transcription factor complex YY1—which promotes neuroblastoma progression by interacting with MYCN [24]—as regulated by ALK. AP-1 transcription factor was also identified as an ALK-regulated phosphoproteins, and AP-1 has been previously found to act downstream of NPM-ALK to promote cell-cycle progression in anaplastic large-cell lymphoma [25]. Together, these results validate our proteomic data given the well-documented effect of ALK inhibition on the downstream transcription factors.

We next performed Gene Ontology (GO) analysis of significantly regulated proteins. The most significant GO categories of the regulated phosphoproteins were similar across all three cell lines. For example, the significantly enriched molecular function GO terms, including GTPase regulator activity (GO:0030695), Ras GTPase activator activity (GO:0005099), and transcription factor binding (GO:0008134), were identified in all three cell lines, albeit with slightly differing but still significant p-values (Supplemental Table S3). The enrichment of these GO terms highlighted RAS proteins as intracellular partners of ALK, transmitting signals from the cell surface to the nucleus.

We next performed KEGG pathway analysis to investigate the potential pathways connecting ALK and downstream transcription factors (Figure 3b). The neurotrophin signaling pathway was significantly overrepresented in the phosphoprotein analysis above. This pathway is known to induce the spontaneous regression of neuroblastoma [26]. These and our results reveal common downstream components shared by the ALK and neurotrophin signaling pathways. For example, phosphorylation levels of IRS2, SHC, ARMS and the intracellular phosphorylation substrates of neurotrophin were markedly reduced after ALK inhibition, suggesting that the potential mechanism by which the neurotrophin pathway induces spontaneous regression is by targeting aberrant ALK. KEGG pathway analysis also identified the MAPK kinase signaling pathway as overrepresented, however, many MAPK pathway components are shared with the neurotrophin signaling pathway (Supplemental Table S3). Additionally, the JNK and p38 MAP kinase pathways were overrepresented to a higher degree than the classic MAPK kinase pathway (Supplemental Figure S4). Taken together, these data demonstrate that the downstream pathways affected by different types of ALK aberration are similar and that ALK promotes cell progression via RAS-JNK-MAP kinase pathways in neuroblastoma.

### JNK signaling is a downstream pathway of ALK in neuroblastoma

**Figure 4:**
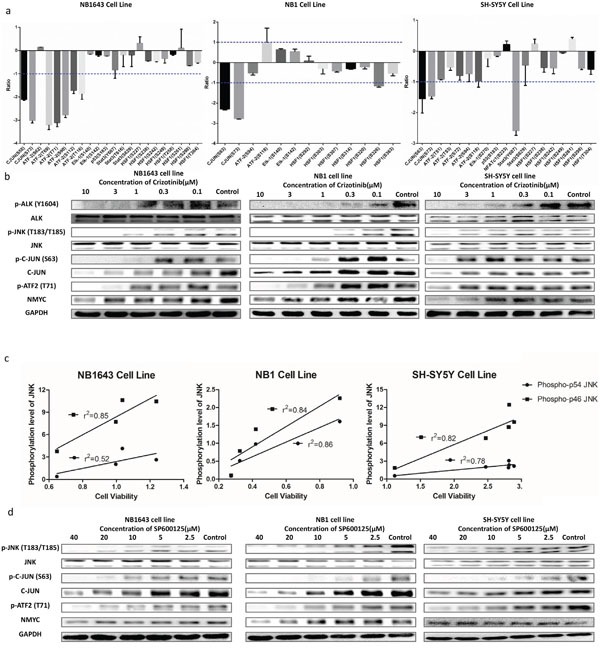
ALK mediates cell viability through JNK signaling pathway **a.** MS quantification result of the phosphorylation sites of the JNK downstream signaling pathways; **b.** Western blot concentration course analysis of cells treated with Crizotinib further confirms the inhibition of JNK signaling pathway; **c.** JNK1/2 phosphorylation level well correlates with cell viability after treating with Crizotinib; **d.** Western blot analysis of cells treated with SP600125 shows similar result of that treated with Crizotinib.

We validated the activation of ALK/JNK signaling by first plotting the phosphorylation levels of JNK substrates following ALK inhibition. Phosphorylation of multiple JNK substrates was dramatically reduced in all three cell lines after treatment with Crizotinib (Figure 4a). Phosphorylation at two sites (Ser63 and Ser73) on the transcription factor c-JUN was decreased by more than ten-fold in all the three cell lines (Figure 4a). These two serine residues are located in the N-terminal transactivation domain of c-JUN and are known to be specific targets of JNK [27], further supporting the notion that ALK promotes neuroblastoma progress via JNK signaling. We then examined the inhibitory effect of Crizotinib *in vitro* by western blotting. The level of phosphorylation of JNK at Thr183 and Tyr185 reflects its activity [28]. The level of JNK phosphorylation was significantly inhibited by Crizotinib in a dose-dependent manner in all three ALK-activated cell lines (Figure 4b), but not in IMR32, a wide type ALK cell line (Supplemental Figure S1b). To further confirm that JNK phosphorylation is dependent on ALK, we used specific siRNA to knock down ALK. Consistently, silencing of ALK significantly reduced cell growth and decreased levels of phosphorylated JNK, c-JUN, and ATF2 (Supplemental Figure S2). Cell viability correlated with JNK phosphorylation after treatment with serially-diluted concentrations of Crizotinib (Figure 4c). We further assessed levels of total c-Jun, phospho-c-JUN (Ser63) (p-c-JUN S63), and phospho-ATF2 (p-ATF2). Levels of p-c-JUN S63 and p-ATF2 were markedly decreased in NB1643 and NB1 cell lines following treatment with 1 μM Crizotinib, whereas 10 μM Crizotinib was required for a similar effect in the SH-SY5Y cell line. Phosphorylation levels of downstream components of the JNK signaling pathway also correlated with cell viability. Additionally, total c-JUN was decreased after Crizotinib treatment, suggesting a potential role for JNK activity in the stabilization of transcription factor AP-1. Together, these data demonstrate that ALK promotes neuroblastoma cell progression via the JNK signaling pathway.

### Inhibition of neuroblastoma cell viability by targeting JNK

**Figure 5:**
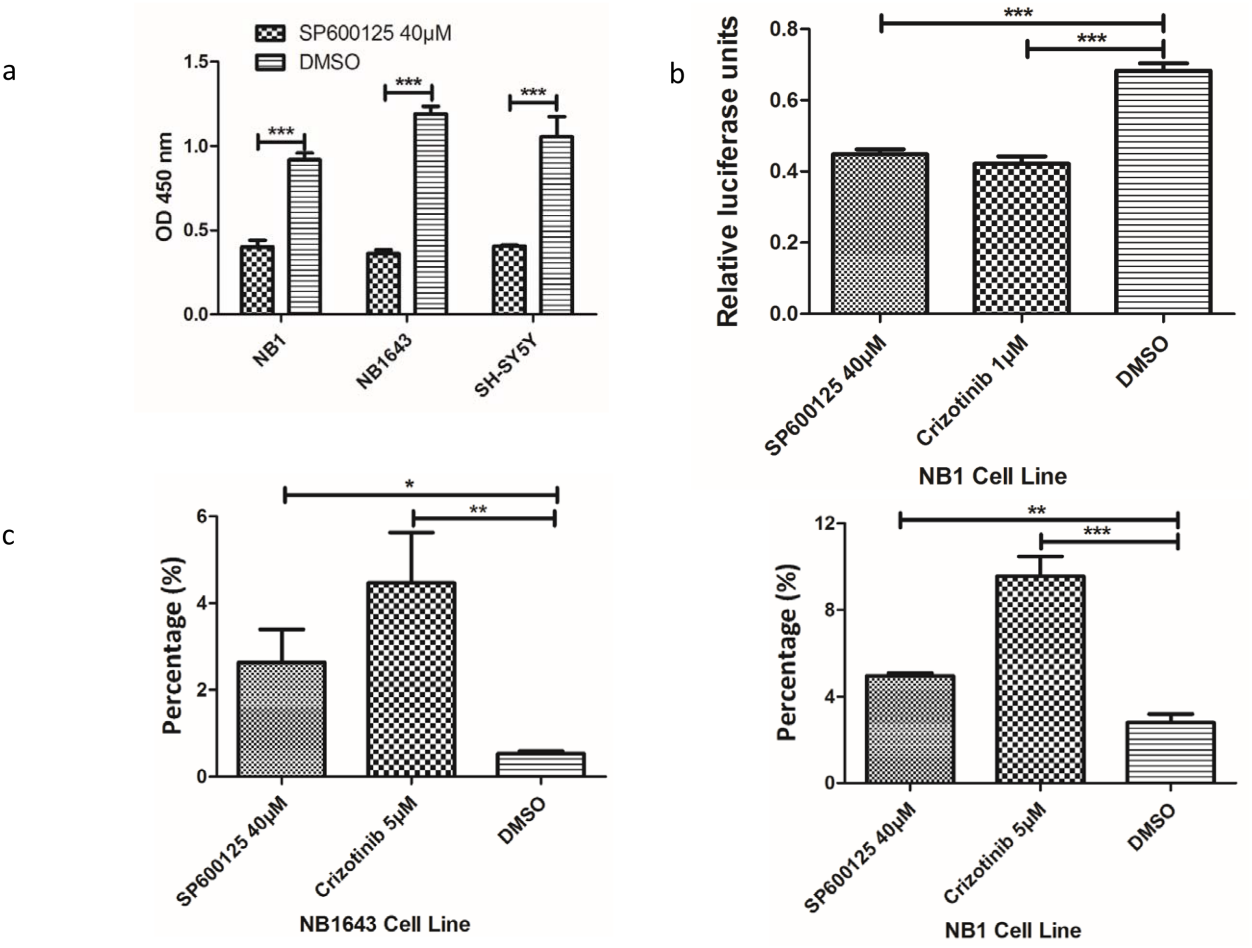
Inhibition of ALK/JNK signaling inactivity reduces cell viability and c-JUN transcriptional activity and increases cell apoptosis **a.** Cell viability is significantly reduced with the treatment of JNK specific inhibitor; **b.** Both ALK and JNK inhibition could significantly inhibit AP-1 promoter activity; **c.** The early apoptosis cells were significantly increased after treatment with JNK inhibitor (SP600125) and ALK inhibitor (Crizotinib)

We further investigated whether cell viability could be inhibited by targeting JNK. Growth of all three cell lines was inhibited by SP600125, a potent and specific small molecular inhibitor of JNK (Figure 5a and Supplemental Figure S5). Further immunoblot analysis confirmed that JNK signal pathway activity was significantly inhibited by SP600125 (Figure 4d). As both ALK and JNK inhibitors significantly reduced the phosphorylation and abundance of c-JUN, a major component of AP-1, we next sought to explore the effect of ALK/JNK inhibition on AP-1 activation using a luciferase reporter assay. NB1 cells transfected with AP-1 luciferase reporter plasmid were incubated with inhibitors of JNK and ALK for 24 h, and a significant decrease in relative luciferase activity was observed (Figure 5b). These findings suggest that aberrant ALK activity promotes neuroblastoma cell progression via JNK signaling.

### ALK/JNK inhibition induces early apoptosis and cell cycle arrest in neuroblastoma

The potential mechanism by which cell growth inhibition is induced by interruption of ALK/JNK signaling was investigated by evaluating the effect of blocking ALK/JNK signaling on both cell apoptosis and the cell-cycle. NB1 and NB1643 cells were incubated with SP600125 and Crizotinib for 24 h and then analyzed using flow cytometry. An obvious increase in early apoptosis was observed after treatment with specific inhibitors, with the early apoptosis fraction significantly augmented in both NB1 and NB1643 cell lines (Figure 5c and Supplemental Figure S6a).

**Figure 6:**
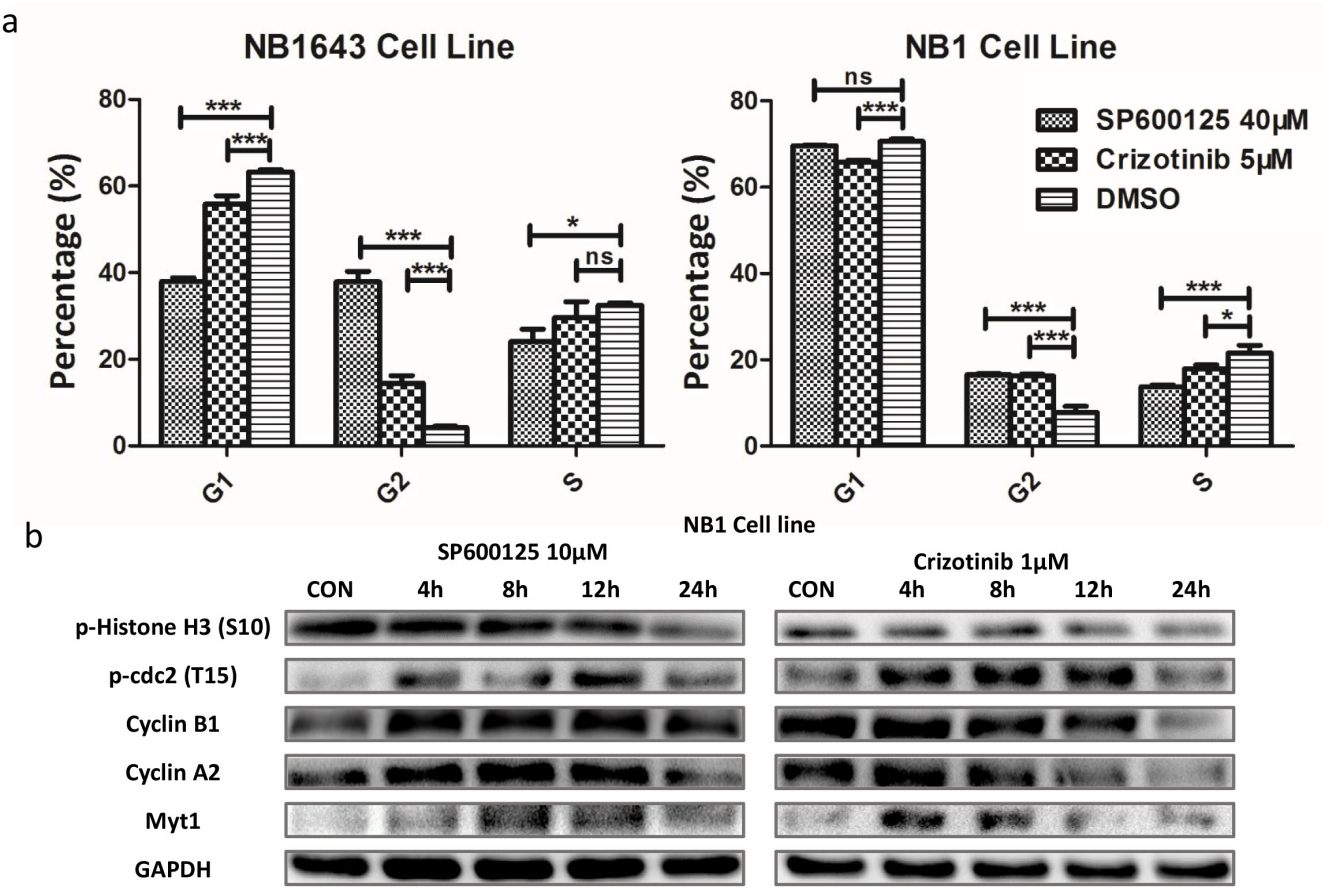
Inhibition of JNK/ALK signaling inactivity reduces cell growth via cell-cycle arrest in neuroblastoma cells **a.** Neuroblastoma cell lines were treated with SP600125 (40 μM), Crizotinib (5μM) and DMSO for 24 hours. A significant change of the proportion of G1/S and G2/M was observed. **b.** The expression of cell cycle protein was obviously changed over time.

Further cell cycle analysis revealed that significant cell cycle arrest was induced by both inhibitors (Figure 6a, Supplemental Figure S6b), indicating that blocking ALK/JNK signaling also contributed to cell cycle arrest. Both inhibitors promoted cell cycle arrest at G2/M, with this fraction increased by more than three-fold for the ALK inhibitor and nine-fold for the JNK inhibitor in NB1643 cells. For NB1 cells, G2/M arrest increased more than two-fold following treatment with both inhibitors. Western blot analysis demonstrated that blocking the ALK/JNK signaling pathway was associated with increased levels of p-cdc2 (T15) [29], which promotes cell mitosis, and up-regulation of cyclin B1. These results suggest that ALK/JNK promotes cell-cycle progression in neuroblastoma by regulating the G2/M checkpoint (Figure 6b).

## DISCUSSION

Cytoplasmic ALK fusion proteins are present in tumors including NSCLC, ALCL [30], and diffuse large B-cell lymphoma [31]. However, aberrant ALK in neuroblastoma typically presents as full-length membrane-bound receptor protein [4–6]. Amplification and point mutation in the tyrosine kinase domain of full-length ALK can result in constitutive activation of ALK [32]. The current lack of understanding surrounding the molecular mechanisms of aberrant ALK activity in neuroblastoma is one of the main obstacles to the clinical application of Crizotinib in neuroblastoma patients. Hence, there is a great need for a more complete understanding of the downstream signaling pathways by which aberrant ALK signaling functions in neuroblastoma.

In this study, we adapted a phosphoproteomics platform to study the global effects of the ALK inhibitor Crizotinib in three neuroblastoma cell lines bearing different types of aberrant ALK activity. To enhance the depth of phosphoproteomics analysis, we employed high pH chromatography and pre-separated the full proteome digestion into 20 fractions followed by phosphopeptide enrichment [33]. This approach allowed identification of more than 19,500 phosphorylation sites from each cell line—of which approximately 10,000 could be quantitatively analyzed—representing the most in-depth phosphoproteome analysis to date [17, 34, 35]. Bioinformatic analysis of phosphoproteomic data revealed that either the GO categories or the KEGG signaling pathways overrepresented by the significantly regulated proteins were similar amongst all three cell lines examined. These results suggest that different types of aberrant ALK promoted cell progression via similar downstream signaling pathways in neuroblastoma. RAS proteins, a class of small GTPase proteins, deliver signals from cell surface receptors to the nucleus, with overactivation of these proteins implicated in carcinogenesis [36]. The activity of RAS proteins is precisely modulated by GTPase-activating proteins, which can increase their rate of GTP hydrolysis. The significant enrichment of Ras GTPase activator activity (GO:0005099) suggested that RAS proteins function as intracellular partners of cell surface receptor ALK to transmit signals from cell surface to the nucleus. Further KEGG pathway analysis revealed the MAPK kinase pathway as a potent substrate of ALK. Rather than the classic MAPK kinase pathway, our data implicate the ALK-RAS-JNK axis as an important oncogenic signaling pathway in neuroblastoma with activated ALK signaling. The stress activated protein kinase pathways—JNK and p38 MAPK—are frequently perturbed in a range of cancers, with members acting as tumor suppressors or oncoproteins in cell type-specific manners [37]. Oncogenic JNK proteins function by phosphorylating JUN, which subsequently activates AP-1 [38]. Our current findings further reveal that inactivation of ALK either by Crizotinib or specific siRNA significantly decreased levels of phosphorylated JNK, JUN, and ATF2. This significantly reduced the activity of transcription factor AP-1. Similar effects were also observed in cell lines treated with JNK inhibitor (Figure 5b). Together, these data clearly demonstrate the oncogenic functions of JNK as a downstream mediator of ALK signaling in neuroblastoma. Therefore, excessive JNK activity could be exploited as a potential biomarker of ALK inhibitor sensitivity in neuroblastoma. Furthermore, JNK inhibition could represent an alternative strategy for the treatment of some neuroblastoma patients.

Distinct from the fusion form of ALK found in NSCLC, aberrant ALK in neuroblastoma presents as intact protein with point mutations or amplification. This confers differential sensitivity to Crizotinib on neuroblastoma [15]. Interestingly, we also identified differences in ALK phosphorylation substrates in neuroblastoma cells. For example, downregulation of phosphorylated STAT3 induces apoptosis in SH-SY5Y cells [PMID: 24789581], but our data demonstrate that this signaling pathway might not be activated in amplified NB1643 (Figure 4a). This may explain the different sensitivities to Crizotinib in these cells.

Given that F1174L-mutated ALK enhances MYCN protein stabilization [39], we further determined the effects of Crizotinib treatment on MYCN regulation. Indeed, MYCN was decreased with ALK inhibition. Furthermore, we found that other types of aberrant ALK had similar effects, especially in the MYCN amplified cell line NB1643 and NB1 (Figure 4d). Together, our data clearly implicate MYCN as a downstream mediator of ALK signaling in neuroblastoma.

Our current study used quantitative phosphoproteomics to provide a global insight to the downstream pathways regulated by different ALK variants in neuroblastoma. Our data clearly demonstrate that JNKs function as common oncogenic proteins in neuroblastoma independent of the ALK variant present. Additionally, either activation or different isoforms of JNKs could promote diverse cellular responses, ranging from the induction of apoptosis to increased survival. One potential future extension of this work is to elucidate the different phosphorylation substrates of JNK1, JNK2, and JNK3 in neuroblastoma, as this may provide valuable information to discern pharmacological effects from toxicological effects. Comparing the effects of Crizotinib and SP600125 on signaling pathway activation (by phosphoproteomics) and their potential targets (by Kinobeads) [40, 41] are also attractive lines of future investigation. Such knowledge may provide clinically valuable pharmacokinetic details that may be used to stratify tumors for ALK-targeted therapy or to predict the response of an individual tumor to such treatment.

## MATERIALS AND METHODS

### Cell lines and cell culture

Human neuroblastoma cell lines NB1643 cell were obtained from Children's Oncology Group. NB1 and SH-SY5Y cell lines were maintained in RPMI 1640 and DMEM/F12 with 10% fetal bovine serum, respectively. NB1643 cell was cultured in IMDM with 20% fetal bovine serum and 1x ITS (5 μg/mL insulin, 5 μg/mL transferrin, 5 ng/mL selenous acid). All cell lines were incubated humidified air supplemented 5% CO_2_ at 37°C. To enable the quantitative phosphoproteomics, SILAC medium supplemented with 10% dialyzed fetal bovine serum (Invitrogen, Darmstadt) were employed to label the cells, where deficient Arginine and Lysine were supplemented with either stable isotope encoded heavy Arginine and Lysine (Euriso-top) or normal Arginine and Lysine for the light.

### Drug treatment and harvesting

Crizotinib (Selleck Chemicals, Houston, USA) stock solution (10 mM) was prepared by dissolving it in DMSO. Cells were treated with 10 times concentration of IC50 Crizotinib for the three cell lines for 4hrs according drug-response curve (5 μM concentration for NB1 and NB1643, and 10 μM concentration for SH-SY5Y, Supplemental Figure S1). The corresponding control groups were incubated with the same concentration of DMSO (0.1%). Cells were washed with phosphate-buffered saline, 50 mM Tris/HCl pH 7.5, 5% Glycerol, 0.8% Nonidet P-40, and lysed with freshly added protease (SIGMAFAST, Sigma-Aldrich, Munich, Germany) and phosphatase inhibitors (Sigma-Aldrich, Munich, Germany). Homogenates were centrifuged at 6000 *g* at 4°C for 10 min followed by ultracentrifugation at 4°C for 1h at 145,000 *g*, supernatants were collected and aliquots were frozen in liquid nitrogen and stored at −80°C until further use. Protein concentration in lysates was determined by the Bradford assay. The extracted proteins were digested by trypsin as previously described [42]. Briefly, cell lysate were reduced with 5 mM dithothreitol (DTT) at 55°C for 30 min and Cys residues were alkylated with 15 mM iodoacetamide (IAA) at room temperature for 30 min in dark. The alkylation reaction was quenched with 30 mM cysteine for 30 min at room temperature. After the alkylation and reduction, protein lysate was diluted four times with 100 mM NH_4_HCO_3_ (pH 8.0) to decrease the urea concentration to 2 M. Trypsin was then added at an enzyme-substrate ratio of 1:100 (w/w). The digestion was subsequently performed at 37°C for 16hrs and then lyophilized for further analysis.

### High pH RPLC fractionation of tryptic peptides and phosphopeptides enrichments

Before enriching phosophopeptodes, the tryptic peptides were separated by high pH RPLC [33]. Briefly, the separation was performed on a Varian SD1 LC system (Agilent Technologies Inc., Folsom, CA) with an Xbridge C18 column (19×150cm, Waters Corp., Milford, MA) using a 70-min gradient from 2% to 40% of buffer B (10 mM ammonium formate/80%ACN, pH8.5) at a flow rate of 10mL/min. The sample was collected into 60 fractions based on equal time intervals (about 10mL/tube), and combined into 20 fractions. Each fraction was lyophilized with SpeedVac.

Phoephopeptides were enriched with customized IMAC beads as previously described [17]. Ion-chelated IMAC beads were prepared from Ni-NTA superflow agarose beads as reported by Ficarro *et al*[43]. Briefly, fractioned peptides were dissolved in binding buffer (80%ACN, 0.1%TFA in ddH_2_O) and incubated with above IMAC beads on vortex for half hour at room temperature. After washing one time with binding buffer and two times with washing buffer (0.1%TFA in ddH_2_O), phosphopeptides were eluted three times by incubating with elution buffer (4% NH_4_OH) for 5min. The elutes were then combined and neutralized with 50 μl of 10% TFA before lyophilized for the following MS analysis.

### Liquid chromatography tandem MS (LC-MS/MS) analysis

Nanoflow LC-MS/MS was performed by coupling an an EASY-nLC 1000 system to a Thermo Orbitrap Elite (Thermo Fisher Scientific, San Jose, CA). Briefly, Tryptic peptides of each fraction were dissolved in solvent A (0.1% formic acid, 2% acetonitrile, 98% H2O) and directly loaded onto a manually packed reversed-phase C18 pre-column (30 mm× 79μm, 5-μm particle size, Dikma, China) for online desalting. Peptide separation was performed using a manually packed reversed phase C18 column (170 mm× 79μm, 3-μm particle size, Dikma, China) coupled to Easy nLC 1000 (Thermo Fisher Scientific, Waltham, MA). Peptides were eluted by using a linear gradient of 8-32% solvent B (0.1% formic acid in 90% acetonitrile and 10% H2O) for 51 min, 32-48% for 5min and 48-80% for 1min and 80% for 3min at a constant flow rate of 300nl/min. The eluted peptides were ionized using NSI source, and analyzed in an Orbitrap Elite mass spectrometer (Thermo Fisher Scientific, Waltham, MA). For full MS spectra, intact peptides with m/z 350-1300 were detected in the Orbitrap at a resolution of 120,000 at *m/z* 400 with automatic gain control (AGC) 1×10^6^. The ten most intense ions were sequentially isolated in the linear ion trap and subjected to collisionally induced dissociation (CID) with a normalized energy of 35%. The dynamic exclusion duration was set to be 60 s, and the precursor ions were isolated by quadrupole with isolation window 1 Da. The fragment ions were analyzed in ion trap with AGC 7,000 at rapid scan mode.

### MS data analysis

The raw spectra data was processed by Maxquant software for peak detection and quantification (v1.4.1.2) [44]. MS/MS spectra data was searched against the Uniprot human database (88,817 sequences) by Andromeda search engine [45] enabling contaminants and the reversed versions of all sequences with the following search parameters: carbamidomethylation of cysteine residues as fixed modification and acetyl (Protein N-term), oxidation (M) and phosphorylation (STY) as variable modifications. Trypsin was specified as the proteolytic enzyme with maximum 2 missing cleavages. After the preliminary search with 20ppm of mass tolerance, the mass accuracy of the precursor ions was decided by the time-dependent recalibration algorithm of Maxquant. Seven ppm was finally used for main search of the precursor ions while fragment ion mass tolerance was set to 0.6 Da. The maximum false discovery rate (FDR) for proteins and peptides was 0.01 and a minimum peptide length of six amino acids was required.

### Bioinformatic analysis

Raw protein abundance values were first normalized using Quantile normalization to stabilize the distributions across the samples (Supplemental Figure S1) [46]. Perseus (v1.4.0.20) was further employed for the further data processing. The contaminate proteins and reverse sequence hits were excluded and only significantly regulated proteins were used for the following analysis. The subcellular localization of the individual proteins was analyzed by querying LOCATE database [20]. Transcription factor prediction was searched against DBDs database (DNA binding domains) [47]. The biological process (BP), molecular function (MF), cellular component (CC), KEGG pathways and UCSC_TFBS (transcription factor binding sites annotation) analysis of the significantly expressed proteins was performed using Database for Annotation, Visualization and Integrated Discovery Bioinformatics Database (DAVID 6.7) [48, 49].

### Western blot analysis

Cells were seeded in 6 well plates until the cell confluency up to 80%, a serial concentration of SP600125 and Crizotinib was employed to treat the cells for 4 hours. After washing twice with cold PBS, the cells were lysed with RIPA (Thermo Scientific, Rockford) lysis buffer containing 5X protein phosphatase inhibitor and PMSF. Protein concentration was determined by BCA kit (Thermo Scientific, Rockford). A 10% SDS-PAGE gel was used for gel electrophoresis. Each lane was loaded equal amounts of proteins. A PVDF membrane was used for protein transfer. Membrane was then blocked using 5% w/v BSA in TBST (Tris-buffered saline, pH 7.6, 0.1% Tween®20) for 1 hour in room temperature prior to incubation with primary antibody using 5% w/v BSA in TBST at 4°C for overnight. Membrane was washed thrice with TBST for 30 min, incubated with appropriate secondary antibody using TBST for 30 min in room temperature, and then washed thrice with TBST for 30 min. Proteins was visualized by electrogenerated chemiluminescence (ECL). Antibodies were purchased from Cell Signaling Technology (Beverly, MA, USA) and used to direct against total JNK (#9252), Phospho-SAPK/JNK (#4668), total c-jun, phospho-c-Jun (Ser63) (#2361), phospho-ATF-2 (Thr71) (#5112), ALK (#3333), phospho-ALK (Tyr1507) (#14678), Cyclin A2 (#4656), phospho-cdc2 (Tyr15) (#4539), Myt1(#4282), phospho-Histone H3 (Ser10) (#3377) and Cyclin B1 (#12231). Anti-GADPH (#2118) were used as a loading control.

### Apoptosis and cell cycle analysis

Apoptosis was analyzed using Annexin V-FITC Apoptosis Detection Kit (BD Biosciences Pharmingen, San Diego, CA) and BD Biosciences FACSCalibur flow cytometry. Briefly, a total of 1.5*10^4^ cells were washed twice with cold PBS and resuspended with binding buffer, then stained with Annexin V-FITC and propidium iodide (PI) for 15 min in the dark. Analyze by flow cytometry within 1 hour. Cell cycle analysis was used PI staining and flow cytometry. 3*10^4^ cells were harvested and fixed with cold 70% alcohol overnight at 4°C. After fix cells overnight, the cells were washed with PBS and resuspended with RNase. After incubation for 30 min at 37°C, PI was added to cells and analyze within 4 hours.

### siRNA transfection

The small interfering RNA (siRNA) specific for ALK was chemically synthesized (Genomeditech, Shanghai, China). The sequence of ALK siRNA was CTGGTCATAGCTCCTTGGAAT. The sequences of ALK siRNA were previously reported [7]. The siRNA transfection was performed by using lipofectamine 2000 (Life Technologies, Waltham, MA) according to the protocol for transfection. 50nM siRNA was used in the NB1 cell line, after transfected for 72 hours, the cells were lysed for western blot analysis.

### Transfection and luciferase reporter assay

When cells reached 80–90% confluent in 24-wells plates, plasmids were incubated in Opti-MEM (Gibco, Waltham, MA) by using lipofectamine 2000 (Life Technologies, Waltham, MA) according to the protocol for transfection. As for NB1 cell, a total of 2.4ul lipofectamine 2000, 5ug pGL4.44[luc2P/AP1 RE/Hygro] vector and 0.2ug Renilla luciferase vector were used in each well. After incubating with SP600125 (40μM) and Crizotinib (1μM) for 24 hours, the cells were lysed and analyzed with Dual-Luciferase Reporter Assay System (Promega, Madison, Wisconsin).

## SUPPLEMENTARY FIGURES AND TABLES








