# Supplementary material for: Phosphoproteomics reveals ALK promote cell progress via RAS/JNK pathway in neuroblastoma
Source: Oncotarget. 2016 Oct 7;7(46):75968–80. doi: 10.18632/oncotarget.12513 (PMC5342791; doi:10.18632/oncotarget.12513)
Supplement: Supplementary file 1 [file oncotarget-07-75968-s001.pdf]

Phosphoproteomics reveals ALK promote cell progress via RAS/JNK pathway in neuroblastoma

SUPPLEMENTARY FIGURES AND TABLES

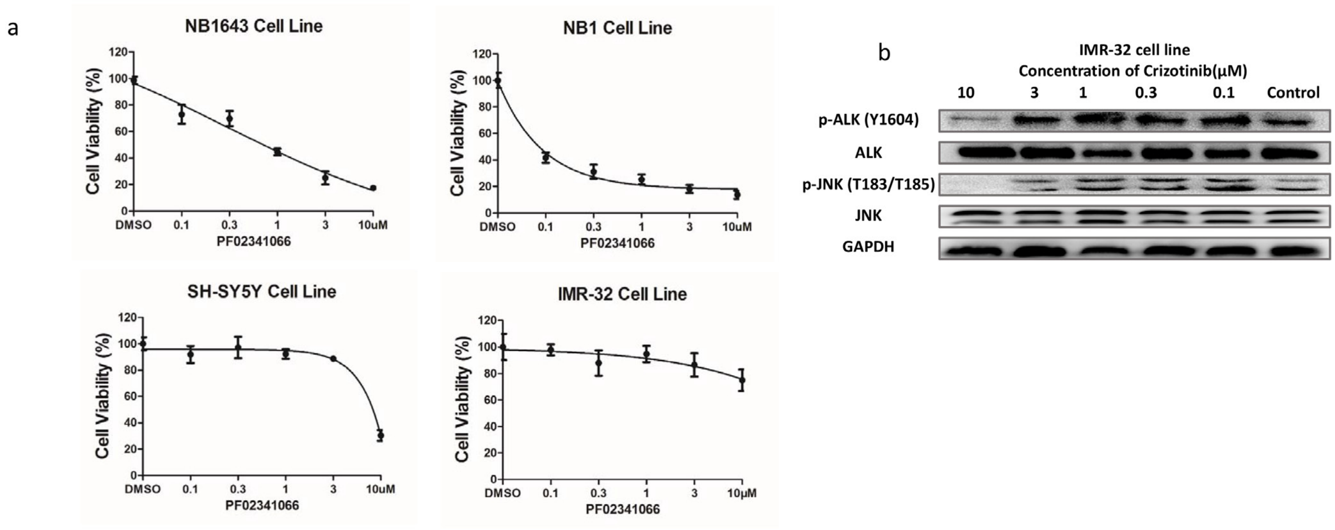

Supplementary Figure S1: a. Dose response measurement of cells incubated with the indicated concentrations of Crizotinib. Cell viability was determined after 72h with CCK-8. **b.** Western blot concentration course analysis of IMR-32 cells treated with Crizotinib for 4hour.

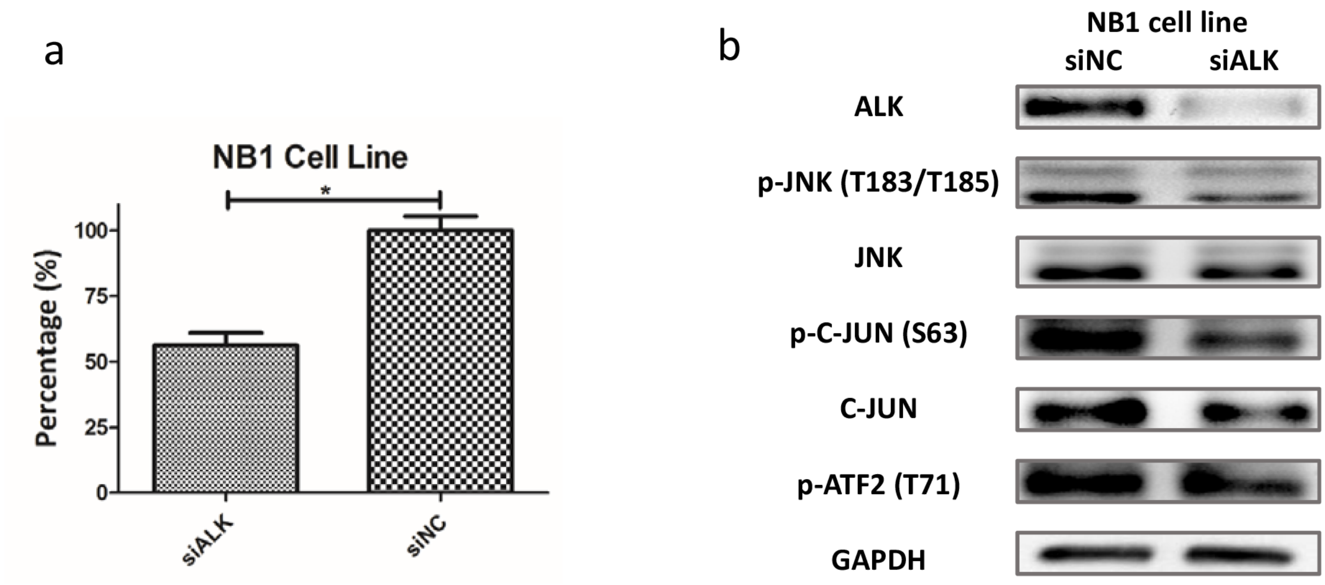

Supplementary Figure S2: a. The viability of NB1 cells at 72 h post-infection with siRNA against ALK was evaluated by CCK-8 assays; **b.** NB1 cells were transfected with siALK or scrambled siRNA for 72h, western blot analysis showed the decreased phosphorylation level of JNK signaling pathway.

a

Total noval p-sites	pS sites	pT sites	pY sites	Class I p-sites	% of Class I p-sites
9693	7384	2059	250	5304	54.72%

b

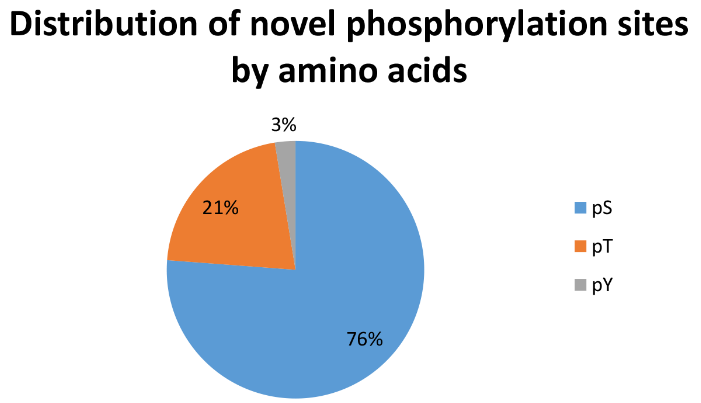

c

Cell lines	Total regulated p-sites	Down regulated p-sites	Up-regulated p-sites	Class I p-sites
NB1643	790	624	166	88.86
NB1	438	337	101	92.47
SHSY5Y	497	340	157	88.73

Supplementary Figure S3: Novel phosphorylation sites identified from three cell line by comparing with the PhosphoSitePlus database. a. Number of the novel phosphorylation sites by amino acid; **b.** Distribution of the novel phosphorylation sites by amino acid; **c.** Number of significantly regulated phosphorylation sites in three cell lines.

Pathway:MAPK signaling pathway
Pathway information generated by KEGG. Stop Blinking

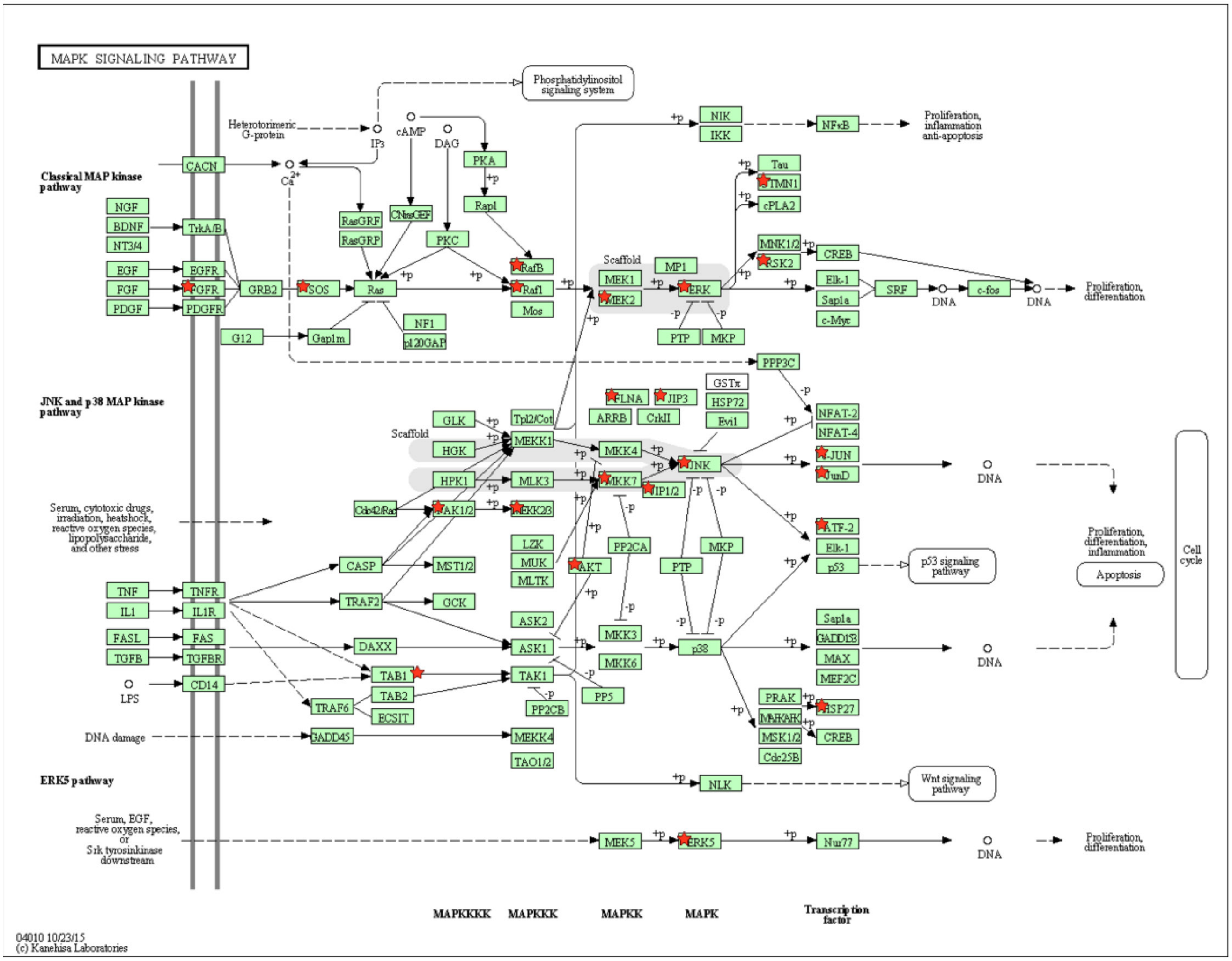

Supplementary Figure S4: MAPK signaling pathway enriched by the significantly regulated phosphorylated proteins in NB1643 cell line, which are marked with red star.

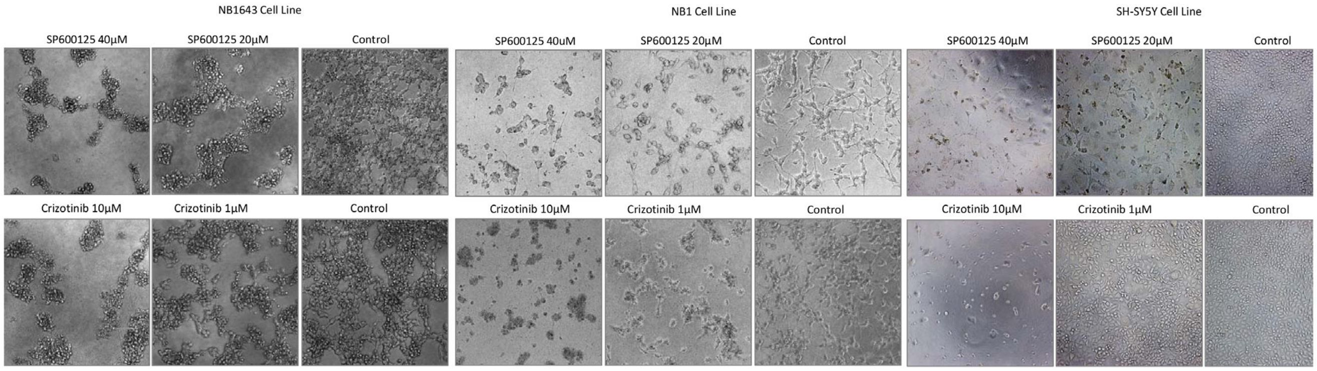

Supplementary Figure S5: Morphological changes and cell number decreases are observed after treatment with different concentration of JNK and ALK inhibitor after 72 hours in all three neuroblastoma cell lines.

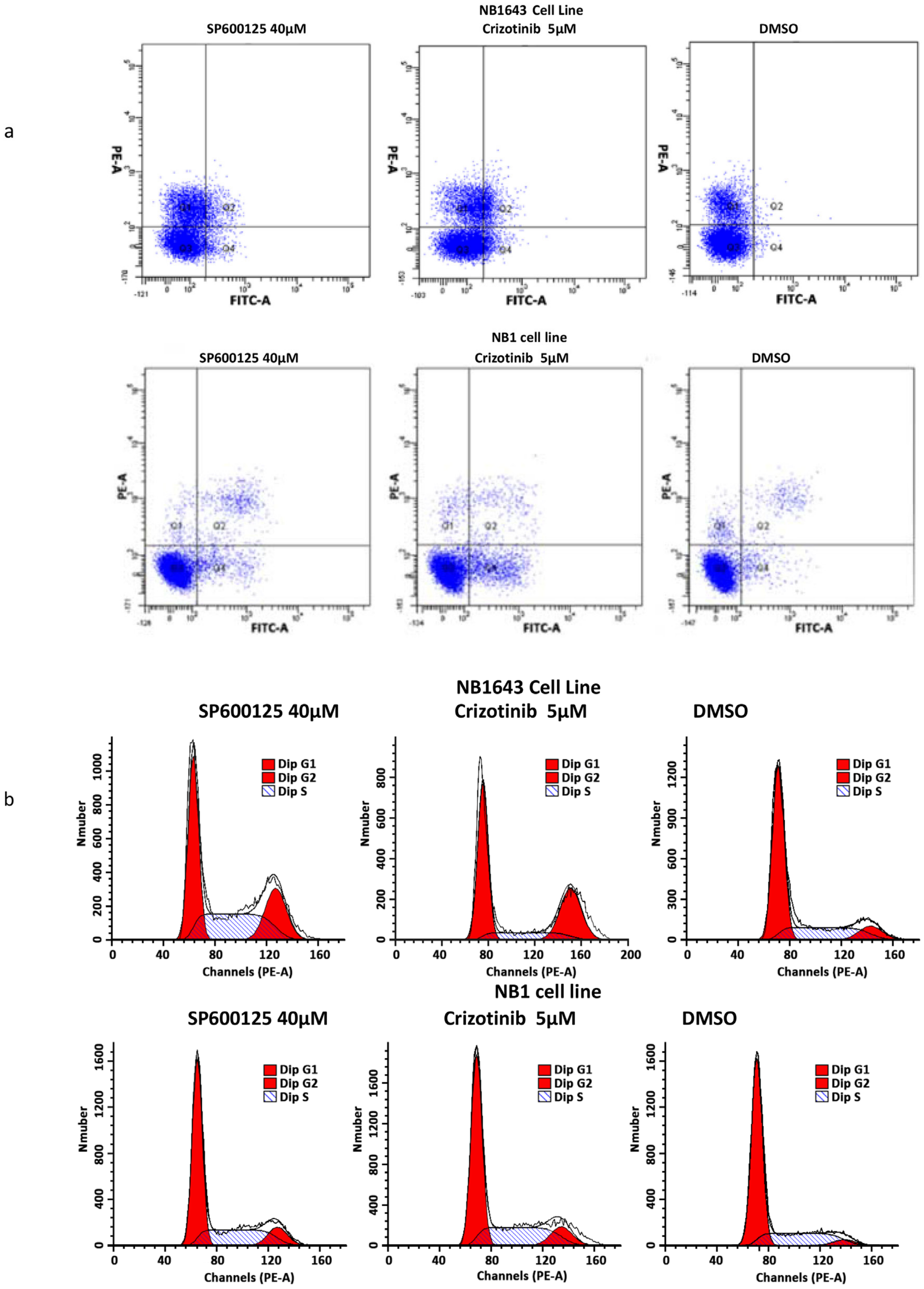

Supplementary Figure S6: Flow cytometry analysis of apoptosis and cell cycle were conducted after treated with SP600125 (40μM), Crizotinib (5μM) and DMSO for 24 hours in NB1 and NB1643 cell line. The percentage of early apoptotic cells treatment with ALK and JNK inhibitors were remarkably higher comparison with the DMSO, besides the cell cycle arrest also was observed.

Supplementary Table S1: List of phosphorylation peptides identified from three cell lines

See Supplementary File 1

Supplementary Table S2: List of transcriptional factors identified from the significantly regulated phosphoproteins

See Supplementary File 2

Supplementary Table S3: GO and KEGG analysis of the significantly regulated phosphoproteins

See Supplementary File 3
